# Supplementary material for: Comparative Genomics of Three Colletotrichum scovillei Strains and Genetic Analysis Revealed Genes Involved in Fungal Growth and Virulence on Chili Pepper
Source: Front Microbiol. 2022 Jan 27;13:818291. doi: 10.3389/fmicb.2022.818291 (PMC8828978; doi:10.3389/fmicb.2022.818291)
Supplement: Supplementary file 1 [file Data_Sheet_1.PDF]

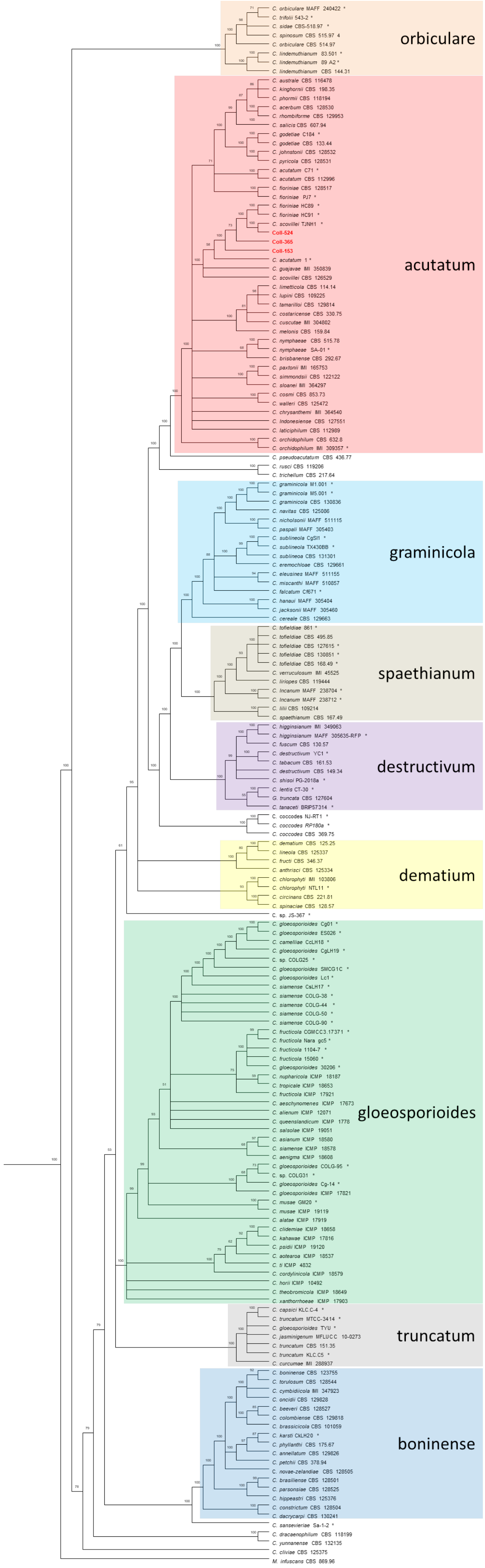

Figure S1. Phylogenetic tree of 173 *Colletotrichum* strains and one *Monilochaetes infuscans* strain showing the phylogenetic relationships among the three *Colletotrichum* strains (Coll-524, Coll-153 and Coll-365) and other *Colletotrichum* species. The phylogeny was constructed by using DNA sequences of five markers (ACT1, CHS1, GAPDH, ITS and TUB2) according to Cannon et al. (2012) with additional 62 *Colletotrichum* strains from NCBI as indicated in Table S1. The 62 strains from NCBI are marked with an asterisk (*). The values at the nodes are Bayesian posterior probability values. Strains belonging to various *Colletotrichum* species complexes are indicated with different color boxes.

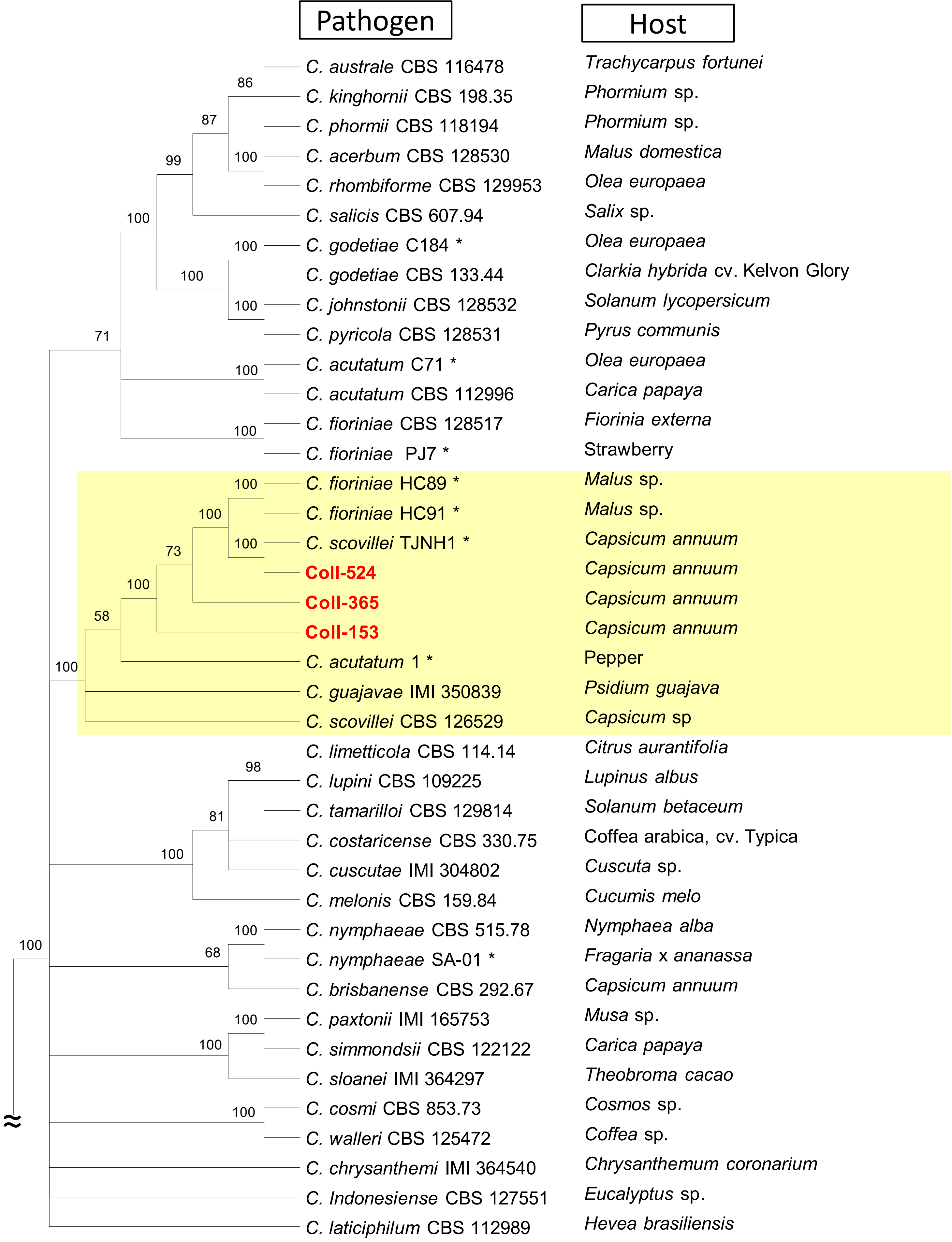

Pathogen
Host
C. australe CBS 116478
Trachycarpus fortunei
C. kinghornii CBS 198.35
Phormium sp.
C. phormii CBS 118194
Phormium sp.
C. acerbum CBS 128530
Malus domestica
C. rhombiforme CBS 129953
Olea europaea
C. salicis CBS 607.94
Salix sp.
C. godetiae C184 *
Olea europaea
C. godetiae CBS 133.44
Clarkia hybrida cv. Kelvon Glory
C. johnstonii CBS 128532
Solanum lycopersicum
C. pyricola CBS 128531
Pyrus communis
C. acutatum C71 *
Olea europaea
C. acutatum CBS 112996
Carica papaya
C. fioriniae CBS 128517
Fiorinia externa
C. fioriniae PJ7 *
Strawberry
C. fioriniae HC89 *
Malus sp.
C. fioriniae HC91 *
Malus sp.
C. scovillei TJNH1 *
Capsicum annuum
Coll-524
Capsicum annuum
Coll-365
Capsicum annuum
Coll-153
Capsicum annuum
C. acutatum 1 *
Pepper
C. guajavae IMI 350839
Psidium guajava
C. scovillei CBS 126529
Capsicum sp
C. limetticola CBS 114.14
Citrus aurantifolia
C. lupini CBS 109225
Lupinus albus
C. tamarilloi CBS 129814
Solanum betaceum
C. costaricense CBS 330.75
Coffea arabica, cv. Typica
C. cuscutae IMI 304802
Cuscuta sp.
C. melonis CBS 159.84
Cucumis melo
C. nymphaeae CBS 515.78
Nymphaea alba
C. nymphaeae SA-01 *
Fragaria x ananassa
C. brisbanense CBS 292.67
Capsicum annuum
C. paxtonii IMI 165753
Musa sp.
C. simmondsii CBS 122122
Carica papaya
C. sloanei IMI 364297
Theobroma cacao
C. cosmi CBS 853.73
Cosmos sp.
C. walleri CBS 125472
Coffea sp.
C. chrysanthemi IMI 364540
Chrysanthemum coronarium
C. Indonesiense CBS 127551
Eucalyptus sp.
C. laticiphilum CBS 112989
Hevea brasiliensis
86
87
99
100
100
100
100
100
71
100
100
100
100
100
100
73
100
58
100
98
81
100
100
68
100
100
100
100

Figure S2. The original host and phylogenetic relationship of strains in the *Colletotrichum acutatum* species complex. This tree is part of the phylogenetic tree in supplementary Figures S1. The original host of each strain is indicated on the right side behind each strain. The clade containing Coll-524, Coll-153 and Coll-365 is highlighted in yellow.

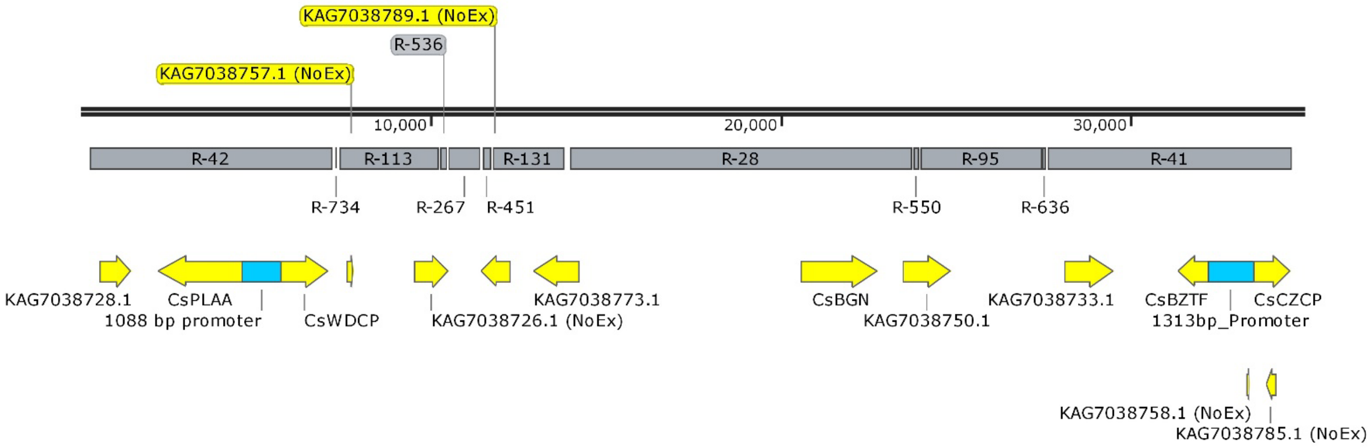

Figure S3. Genetic map of the 14 ORF-Vrem in scaffold 17 of strain Coll-365. The gray bars with R-number indicated the removed fragments in Coll-365 compared to Coll-524. The 14 genes located in the 34.9-kb fragment of scaffold 17 are genes KAG7038728.1, *CsPLAA, CsWDCP*, KAG7038757.1, KAG7038726.1, KAG7038789.1, KAG7038773.1, *CsBGN*, KAG7038750.1, KAG7038733.1, *CsBZTF*, *CsCZCP*, KAG7038758.1 and KAG7038785.1. NoEx: Expression data are non-available.

A

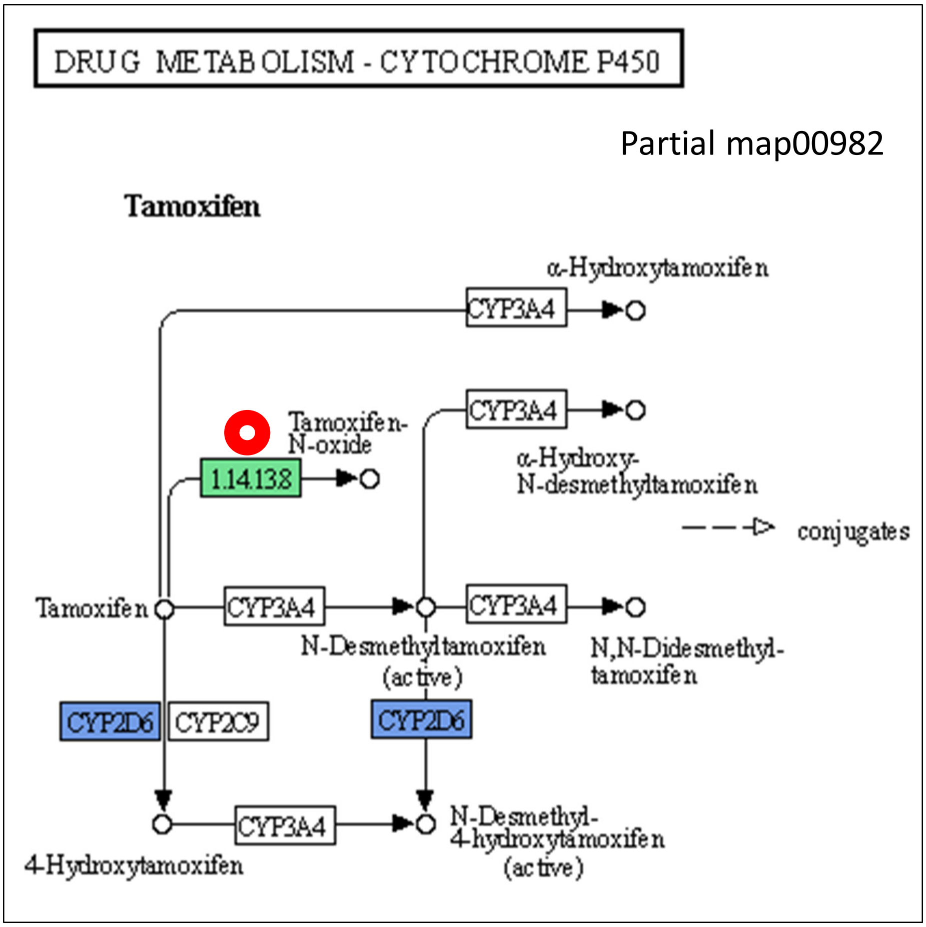

B

KEGG EC number	Coll-524 excess copy of ORF	Orthogroup	Copy number		
			Coll-524	Coll-153	Coll-365
ec:1.14.13.8 monooxygenase	KAG7038404.1<br>KAG7038417.1	OG0000019	6	4	4
	KAG7038482.1	OG0000630	2	1	1
	KAG7038486.1	OG0014590	1	1	0

Figure S4. Drug metabolism – cytochrome P460 KEGG pathway (A) and the gene number variations of monooxygenase among the three strains (B). (A) The drug metabolism KEGG map shown here is a partial map of map00982. (B) The gene number differences of monooxygenase among the three strains were based on the investigation of scaffolds 17, 19, 20 and 22. The positions of monooxygenase in the drug metabolism – cytochrome P450 pathway are indicated with red circles in panel A.

A

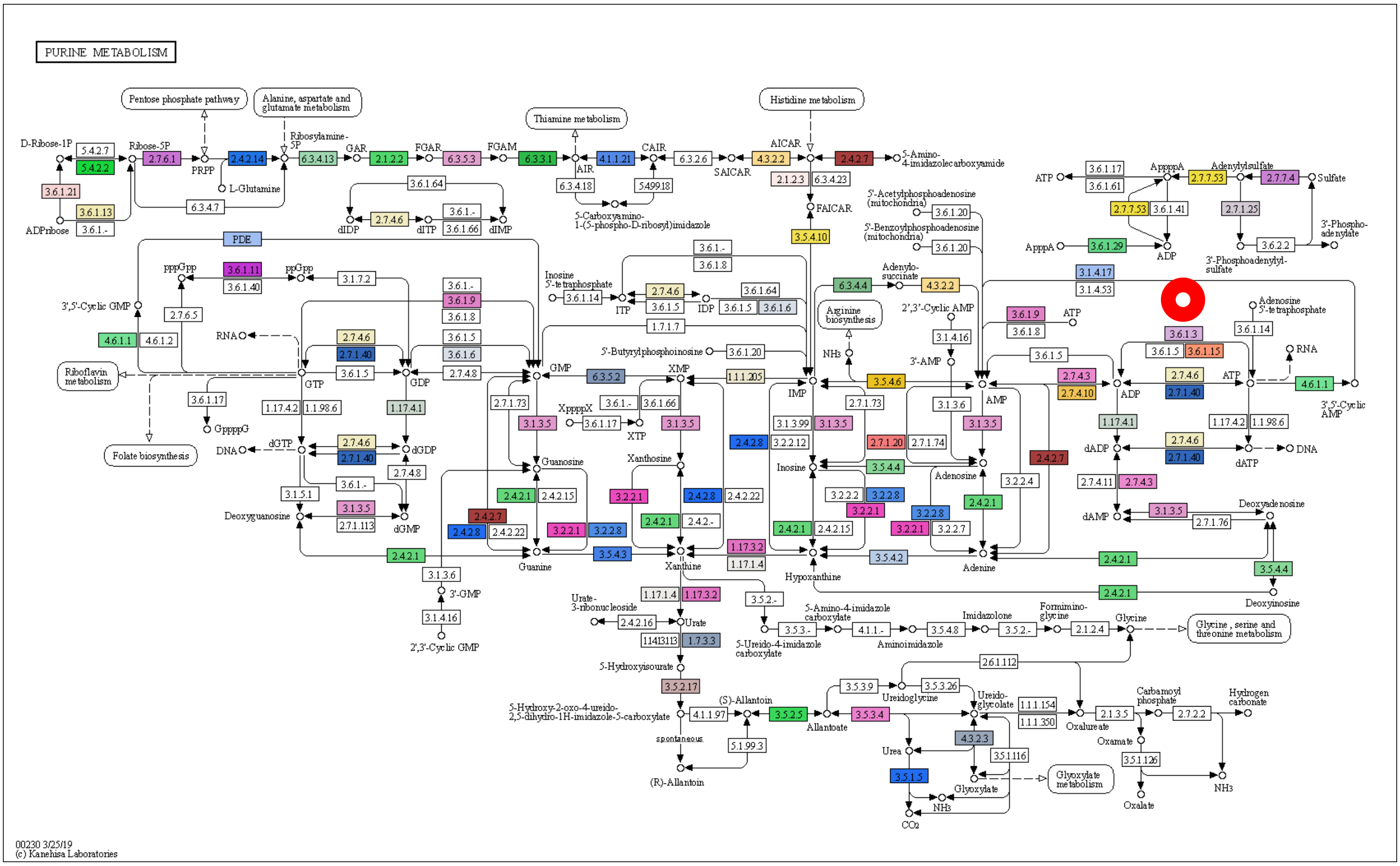

B

KEGG EC number	Coll-524 excess copy of ORF	Orthogroup	Copy number		
			Coll-524	Coll-153	Coll-365
3.6.1.3 - adenylpyrophosphatase	KAG7038453.1	OG0000571	2	1	1

Figure S5. Purine metabolism KEGG pathway (A) and the gene number variations of adenylpyrophosphatase among the three strains (B). The position of the adenylpyrophosphatase in the purine metabolism pathway is indicated with a red circle in panel A.

A

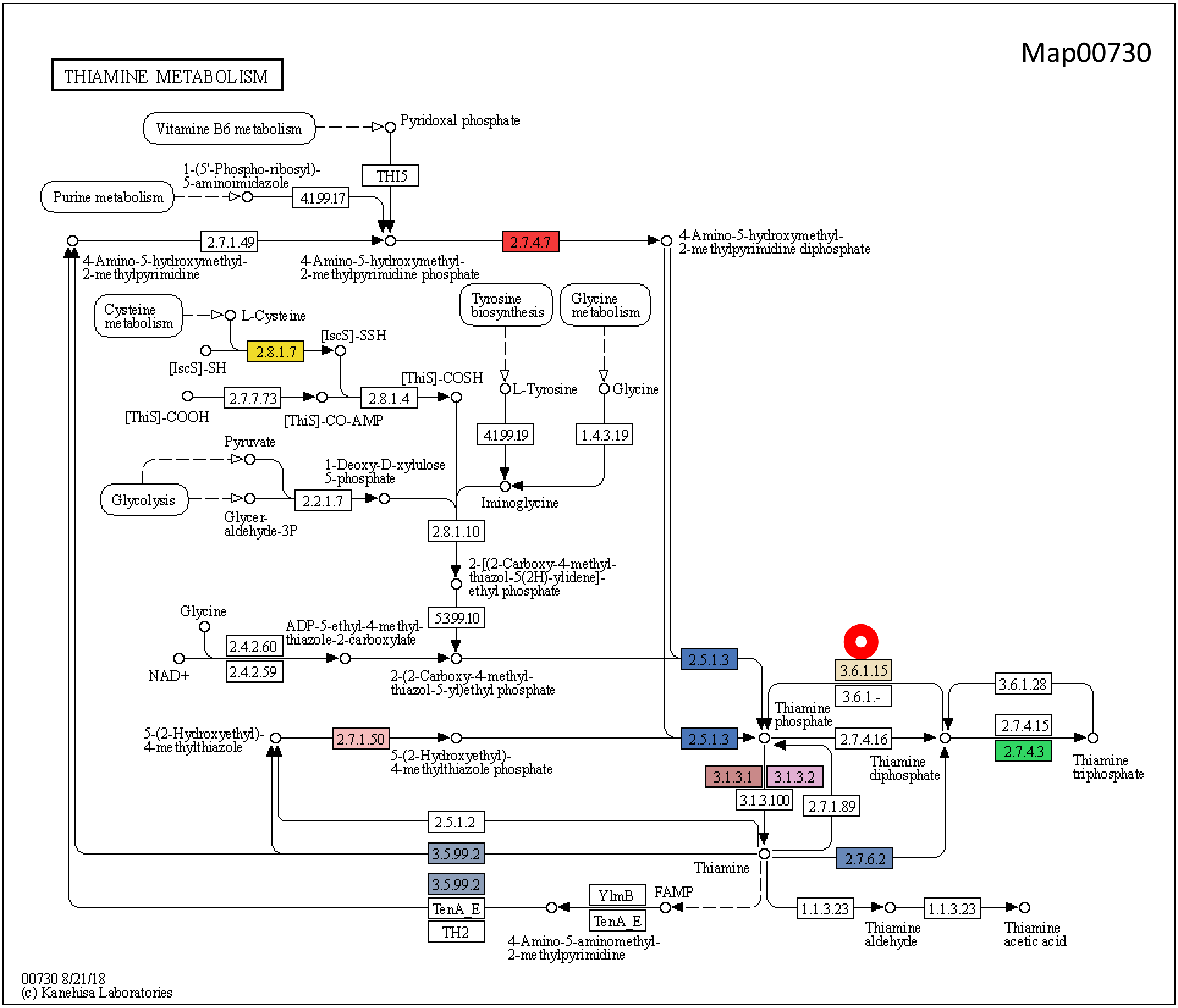

B

KEGG EC number	Coll-524 excess copy of ORF	Orthogroup	Copy number		
			Coll-524	Coll-153	Coll-365
ec:3.6.1.15 phosphatase	KAG7038453.1	OG0000571	2	1	1

Figure S6. The thiamine biosynthesis KEGG pathway (A) and the gene number variations of phosphatase among the three strains (B). The position of the phosphatase in the thiamine biosynthesis pathway is indicated with a red circle in panel A.

A

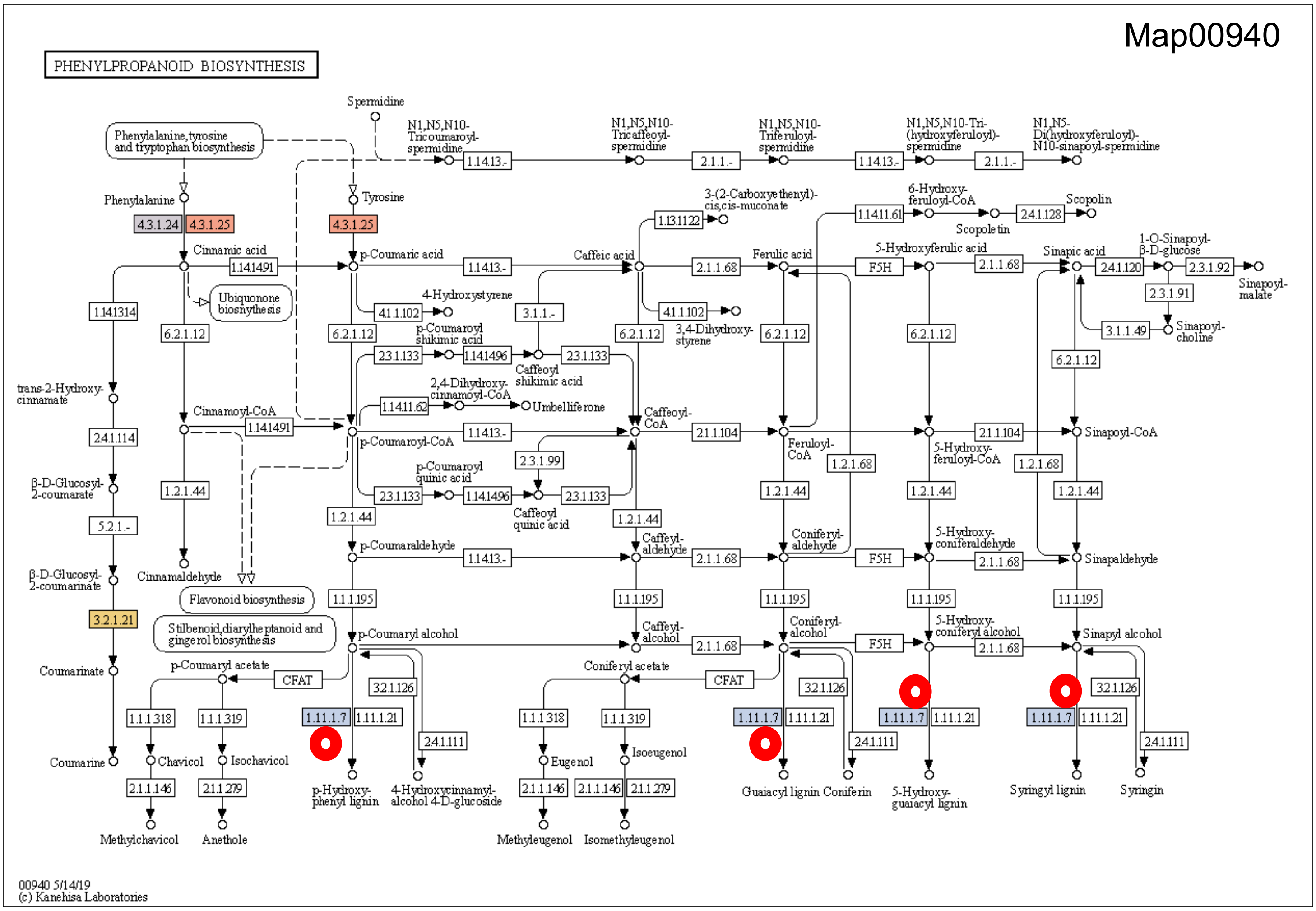

B

KEGG EC number	Coll-524 excess copy of ORF	Orthogroup	Copy number		
			Coll-524	Coll-153	Coll-365
ec:1.11.1.7 lactoperoxidase	KAG7038733.1	OG0014551	1	1	0

Figure S7. Phenylpropanoid biosynthesis KEGG pathway (A) and the gene number variations of lactoperoxidase among the three strains (B). The positions of the lactoperoxidase in phenylpropanoid biosynthesis pathway are indicated with red circles in panel A.

A

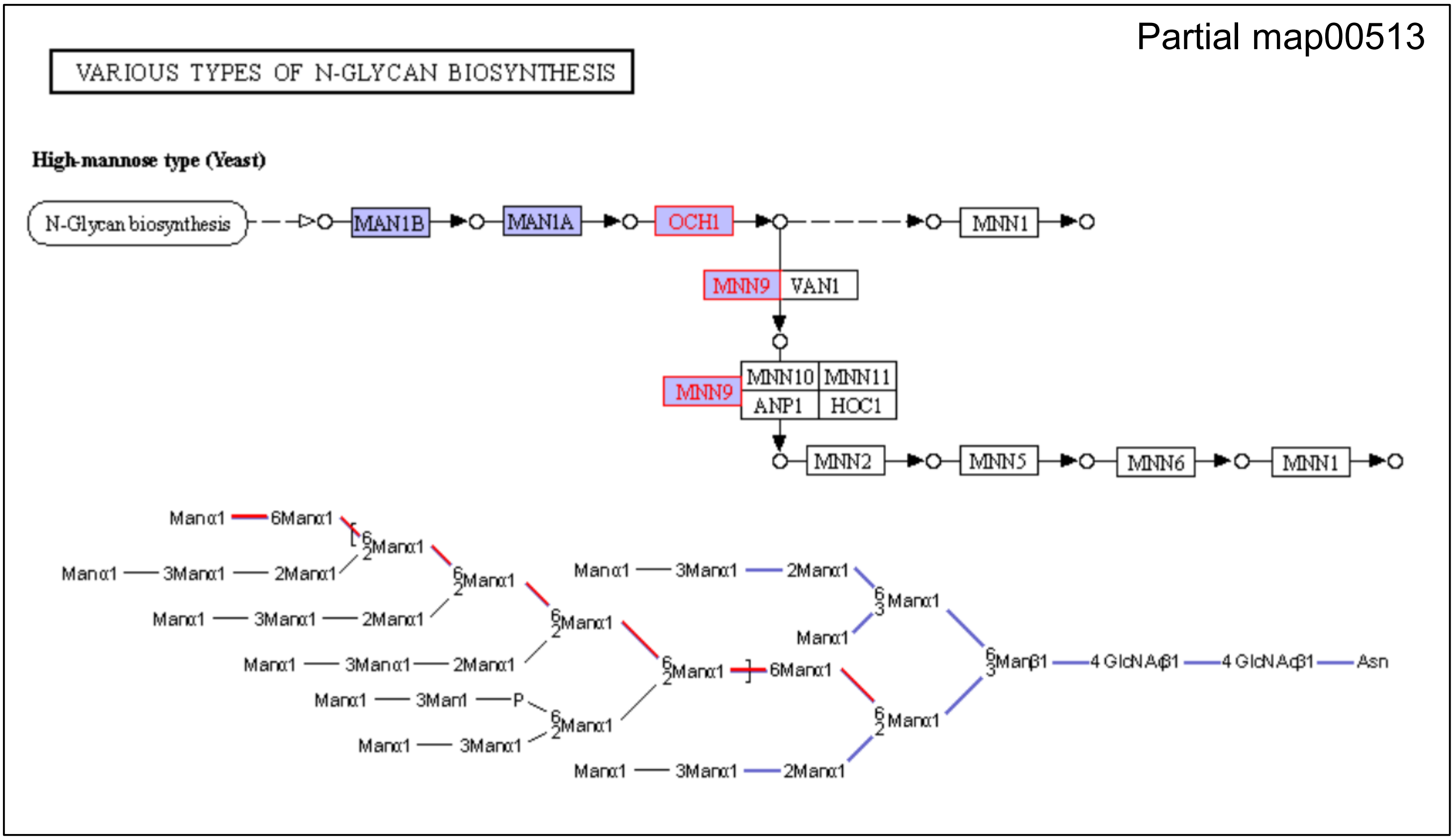

B

KEGG EC number	Coll-524 excess copy of ORF	Orthogroup	Copy number		
			Coll-524	Coll-153	Coll-365
ec:2.4.1.232 alpha-1,6-mannosyltransferase	KAG7038441.1	OG0000642	2	1	1

Figure S8. N-glycan biosynthesis KEGG pathways (A) and the gene number variations of alpha-1,6-mannosyltransferase among the three strains (B). (A) The N-glycan biosynthesis KEGG map shown here is a partial map of map00513. (B) The gene number differences of 1,6-mannosyltransferase among the three strains were based on the investigation of scaffolds 17, 19, 20 and 22. The positions of the 1,6-mannosyltransferase in the purine metabolism pathway are indicated with red boxes and lines in panel A.

Coll-524 DDE-1 containing locus (1891 bp)

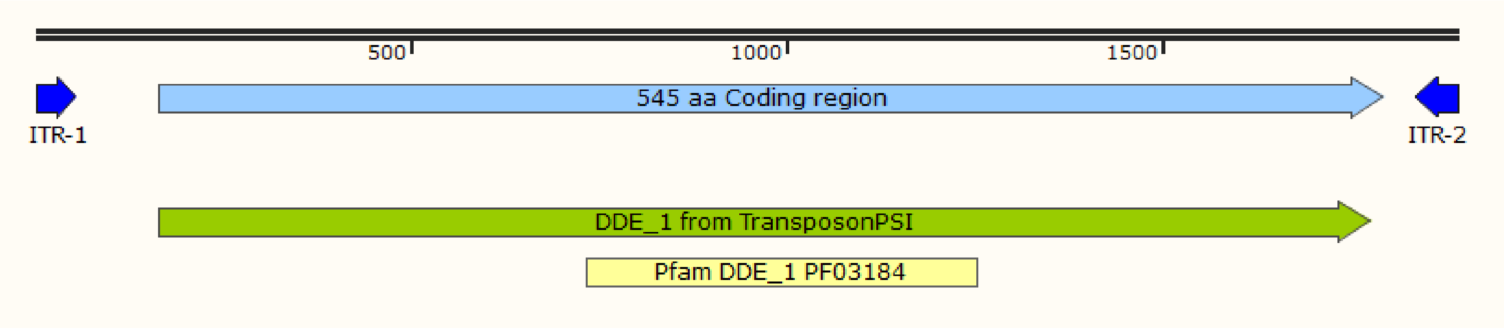

Figure S9. The complete Coll-Fot1 (1891 bp) of strain Coll-524 consists of a coding region and two inverted terminal repeats (ITRs). The DDE_1 containing region provided by TransposonPSI is highlighted with a green arrow. The coding region of 545 aa sequence was identified by SnapGene and is highlighted with a light blue arrow. The yellow bar indicates the DDE_1 domain resulted from InterPro analysis. Two ITRs with 55 bp in length are provided by RepeatModeler and is highlighted as a blue arrow.

A

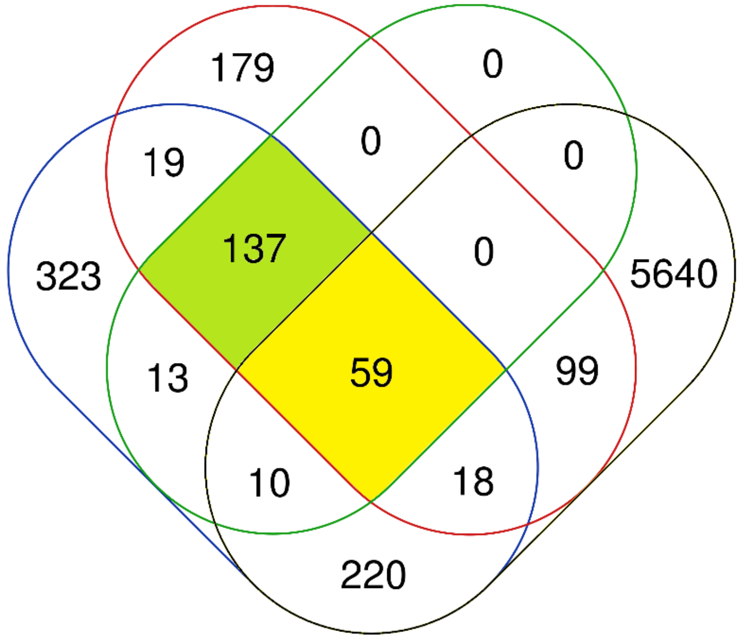

■ : ORF-V of Coll-524 to 153 and/or 365 (799 genes)
■ : Ortholog variation of Coll-524 to 153 and/or 365 (511 genes)
■ : Coll-524 ORFs with <50% coverage to 153 and/or 365 (219 genes)
■ : Coll-524 genes predicted in six pathogenicity related categories, in which PHI was selected with E-value < 10e-50 (6046 genes)

B

Categories		Coll-153 Coll-365	Coll-365
Effector		26 (1)	8
CAZyme			
	GH31	1	0
	AA1	1 (1)	0
	GH17	0	1 (1)
TF		4	1 (1)
KEGG pathway		7	1
PKS cluster in scaffold 18 (including 2 effectors)		7	0
PHI (E-value < 10e-50)		2	1 (1)
Sum		47	12

Figure S10. Venn diagram of four selected gene groups (A) and the clustered 59 genes distribution in six function categories (B). The four selected gene groups were indicated in the bottom of panel A. In panel B, the numbers of genes disappeared in both Coll-153 and Coll-365, and in Coll-365 only were presented. The number in parentheses indicates gene numbers selected for gene functional transformation assay.

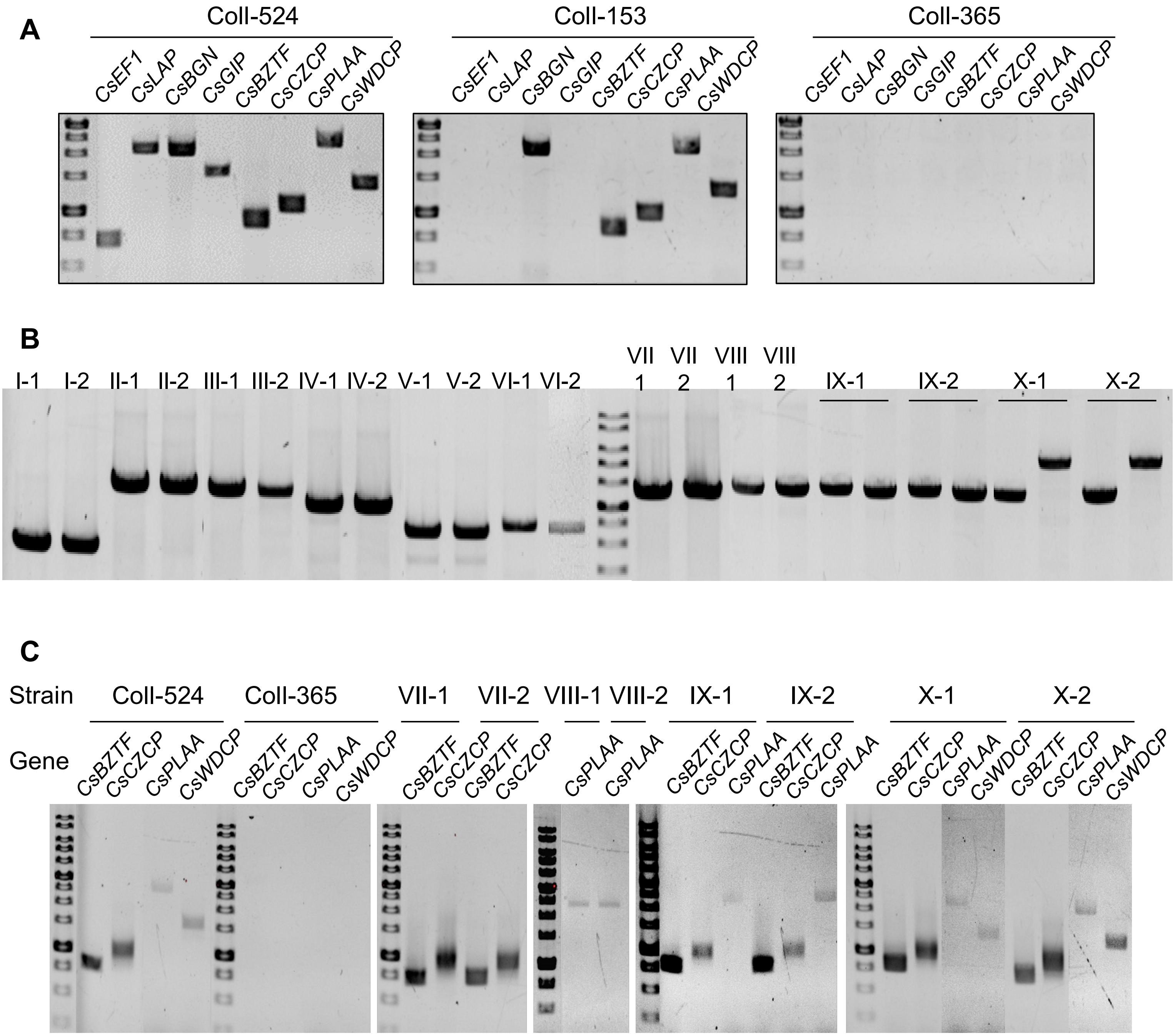

Figure S11. Verification of gene presence in strains Coll-524, 153 and 365 by PCR (A), and confirmation of transgenic strains with regular PCR (B) and RT-PCR (C) for target gene transformation in strain Coll-365.

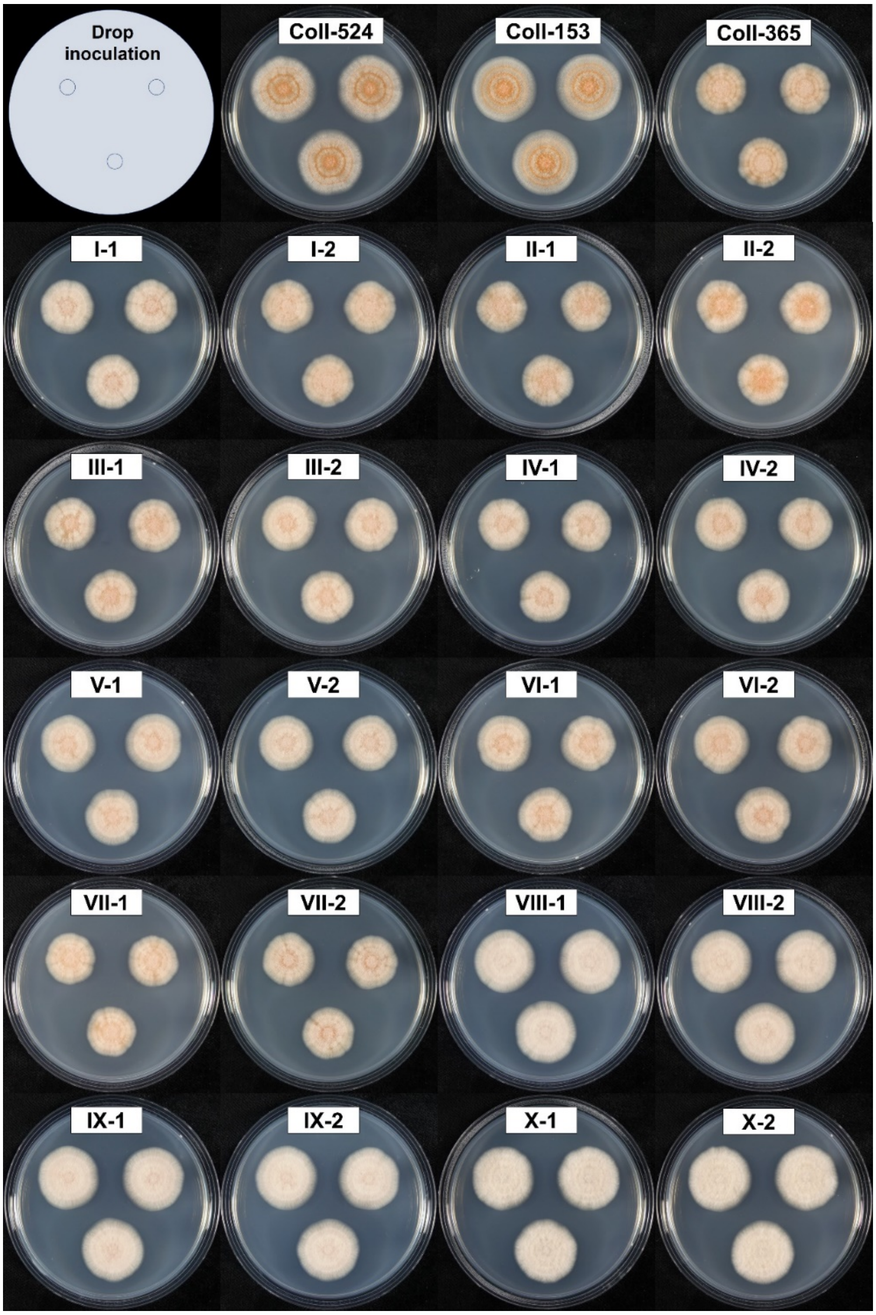

Figure S12. Colony morphology of strains Coll-524, Coll-153, Coll-365 and transgenic strains of Coll-365 on MS agar medium for 5 days at 25℃ under 12 h light. For inoculation, three drops of spore suspension of each strain were dropped on each medium plate as shown at the top left corner.
