# Supplementary material for: Comparative Genomics of Three Colletotrichum scovillei Strains and Genetic Analysis Revealed Genes Involved in Fungal Growth and Virulence on Chili Pepper
Source: Front Microbiol. 2022 Jan 27;13:818291. doi: 10.3389/fmicb.2022.818291 (PMC8828978; doi:10.3389/fmicb.2022.818291)
Supplement: Supplementary file 3 [file Data_Sheet_3.DOCX]

**Supporting Tables**

**Table S1.** The information for all the five maker genes (*ACT1*, *CHS1*, *GAPDH*, *ITS* and *TUB2*) used in the phylogenetic analysis in Figure 2 and Figure S1 and S2. (Please see the excel file.)

**Table S2.** The accession numbers of genes analyzed in this research.

| Gene name | Accession number |
| --- | --- |
| *CsWDCP* | KAG7038739.1 |
| *CsPLAA* | KAG7038764.1 |
| *CsCZCP* | KAG7038737.1 |
| *CsBZTF* | KAG7038776.1 |
| *CsBGN* | KAG7038747.1 |
| *CsEF1* | KAG7049521.1 |
| *CsLAP* | KAG7039340.1 |
| *CsGIP* | KAG7038365.1 |

**Table S3.** Primers used in this study.

| Purpose | Name | Sequence |
| --- | --- | --- |
| Gene confirmation | CsWDCP F | ATGCCCCTCCCCAGCGCAAAC |
|  | CsWDCP R | CTAGTCCACGTTACGTTCAGCAGT |
|  | CsPLAA F | ATGACGACAGACTTCAAGCTATCCGCT |
|  | CsPLAA R | TCACAGCTGTTGGAGAGCTTCT |
|  | CsEF1 F | ATGAGGCGAAAGAAACTTACAGAA |
|  | CsEF1 R | CTAGCCCCAGATCTCGCACTTTG |
|  | CsLAP F | ATGACGTTTTTCAAAAGACTCACAGAGT |
|  | CsLAP R | TTATCTCCTCCCGTGGAATCCAA |
|  | CsBGN F | ATGCCGCGATACGACTTTGAAG |
|  | CsBGN R | TCAGACCGTCGTGCCGCCGCA |
|  | CsGIP F | ATGGCCAAGCGACTATCGCCAGT |
|  | CsGIP R | TTACTCTCGTAGAGCCTTGCATATG |
|  | CsBZTF F | ATGTGCGCCGCATCATCATC |
|  | CsBZTF R | TTACGGCTTAGTTTCCTTACGCATGAC |
|  | CsCZCP F | ATGAATTGCCTCAAGACCCGC |
|  | CsCZCP R | CTTTTCGCAGTACGCTAACAGTTCT |
| Gene complementation | CsWDCP flanking F | GGCAATCTGTGATGGAGGTGG |
|  | CsWDCP flanking R | AACTGAACTAGTAGAGCCGGCATCTCA |
|  | CsPLAA flanking F | GAAGTCAAGGAGGGTCAGTGA |
|  | CsPLAA flanking R | TGACCAGGACGTGCTTCGTTG |
|  | CsEF1 flanking F | GTAGGAGAAGATGACTCTTGGCAGG |
|  | CsEF1 flanking R | AGGAGTTGCTTGGCAAATGCTTGA |
|  | CsLAP flanking F | GTTGCCTACGGCATTCTGGGATCAC |
|  | CsLAP flanking R | GGATTGCAGGTTCAGCGCGTAT |
|  | CsBGN flanking F | AGCCTTGTCTTCAGCATCTCTG |
|  | CsBGN flanking R | AGGTCAAGAGCCCAACCACT |
|  | CsGIP flanking F | GGCTGCCATGACATCCACTC |
|  | CsGIP flanking R | GCATCCGAAGCGTGTCAGTC |
|  | CsBZTF flanking F | ACGGTGGACCATGGAGGATCTGC |
|  | CsBZTF flanking R | ACACGTACCTAAGAGCAGCGAAATG |
|  | CsCZCP flanking F | ACGGCTGCAGACGTGGGTT |
|  | CsCZCP flanking R | CGAGAATGGCATTCCGATCGT |
| Plasmid construction | pBS noMCS R | GTACCCAATTCGCCCTAT |
|  | pBS noMCS F | CGGTGGAGCTCCAGCTTT |
|  | Phle-ri F | ATGGCCAAGTTGACCAGTGC |
|  | Phle-ri R | TCAGTCCTGCTCCTCGGCCA |
|  | TrpC F ecoRV | TCGGATATCTGATATTGAAGGAGCATTTTTTGGG |
|  | NosTer F swaI | TCGATTTAAATGCAGATCGTTCAAACATTTGGCAA |

**Table S4.** Genome mapping of Coll-153 and Coll-365 to Coll-524 analyzed with CLC to provide the datasets of DNA insertion, deletion, removed, and misc. difference in the genomes of Coll-153 and Coll-365.

| **Variation Datasets** | **No. of variation sites or nucleotides** | |
| --- | --- | --- |
|  | **Coll-153** | **Coll-365** |
| Insertion |  |  |
| Insertion sites | 241 | 361 |
| Insertion bps | 338 | 506 |
| Deletion |  |  |
| Deletion sites | 302 | 451 |
| Deletion bps | 417 | 618 |
| Misc. difference^a^ |  |  |
| Misc. difference sites | 7,411 | 9,833 |
| Misc. difference in bps | 9,179 | 12,218 |
| Removed |  |  |
| Removed sites (N^b^<20% of the removed region) | 1,623 | 731 |
| Removed bps (N^b^<20% of the removed region) | 991,585 | 1,179,624 |

^a^, the dataset of Misc. difference consisted of single nucleotide polymorphism (SNP) and polymorphism with more than one nucleotide which was named as multiple nucleotide polymorphism (MNP) in this study.

^b^, N indicates gaps in scaffolds.

**Table S5.** Large DNA fragment removal statistic of strains Coll-153 and Coll-365.

| **DNA removal** | **Coll-153** | **Coll-365** |
| --- | --- | --- |
| ≧20 kb | 0 | 4 |
| ≧10 kb | 1 | 21 |
| ≧5 kb | 7 | 41 |
| ≧1 kb | 325 | 175 |
| <1 kb | 1290 | 490 |
| Sum | 1623 | 731 |

**Table S6.** Orthogroup types and distributions of *Colletotrichum scovillei* strains Coll-524, Coll-153 and Coll-365.

| Numbers of strains in orthogroup | Descriptions of Orthogroups | Numbers of orthogroups  (numbers of genes) | | |
| --- | --- | --- | --- | --- |
|  |  | Coll-524 | Coll-153 | Coll-365 |
| 3 | Single-copy orthogroups | 13,779  (13,779) | 13,779  (13,779) | 13,779  (13,779) |
|  | Multi-copy orthogroups with same numbers | 502  (1,167) | 502  (1,167) | 502  (1,167) |
|  | Multi-copy orthogroups with different numbers | 128  (272) | 128  (192) | 128  (194) |
| 2 | Orthogroups in Coll-524 and Coll-153 | 92  (94) | 92  (92) | - |
|  | Orthogroups in Coll-524 and Coll-365 | 33  (33) | - | 33  (33) |
|  | Orthogroups in Coll-153 and Coll-365 | - | 94  (94) | 94  (94) |
| 1 | Strain-specific orthogroups | 17  (85) | 4  (53) | 4  (56) |
| Sum of orthogroups | | 14,551  (15,430) | 14,599  (15,377) | 14,540  (15,323) |
| Unassigned genes | | (196) | (55) | (64) |

**Table S7.** Functional category statistics of the three *Colletotrichum scovillei* genomes.

| Categories | Gene numbers (% of total genes) | | | | | |
| --- | --- | --- | --- | --- | --- | --- |
|  | Coll-524 | | Coll-153 | | Coll-365 | |
| Effector | 1,857 | (11.9) | 1,811 | (11.7) | 1,810 | (11.8) |
| CAZyme | 664 | (4.2) | 661 | (4.3) | 663 | (4.3) |
| SMURF | 465 | (3.0) | 463 | (3.0) | 472 | (3.1) |
| TF | 349 | (2.2) | 339 | (2.2) | 336 | (2.2) |
| KEGG | 1,789 | (11.4) | 1,787 | (11.6) | 1,780 | (11.6) |
| PHI |  |  |  |  |  |  |
| Increased virulence | 570 | (3.6) | 556 | (3.6) | 561 | (3.6) |
| Reduced virulence | 6,886 | (44.1) | 6,801 | (44.1) | 6,789 | (44.1) |
| Loss of pathogenicity | 887 | (5.7) | 874 | (5.7) | 875 | (5.7) |
| Lethal | 504 | (3.2) | 502 | (3.3) | 502 | (3.3) |
| Mixed results | 616 | (3.9) | 616 | (4.0) | 613 | (4.0) |
| Sum of PHI | 9,463 | (60.6) | 9,349 | (60.6) | 9,340 | (60.7) |

**Table S8.** Functional category statistics of the multi copy orthogroups with different gene numbers for the three *Colletotrichum scovillei* genomes.

| Categories | Coll-524 | Coll-153 | Coll-365 |
| --- | --- | --- | --- |
| Effector | 17 | 9 | 9 |
| CAZyme | 12 | 8 | 9 |
| SMURF | 14 | 8 | 8 |
| TF | 12 | 5 | 6 |
| KEGG | 36 | 33 | 30 |
| PHI |  |  |  |
| Increased virulence | 2 | 0 | 0 |
| Reduced virulence | 94 | 65 | 66 |
| Loss of pathogenicity | 10 | 8 | 7 |
| Lethal | 0 | 0 | 0 |
| Mixed results | 7 | 4 | 4 |
| Mixed functions with reduced virulence | 2 | 2 | 2 |
| Mixed functions with loss of pathogenicity | 2 | 1 | 2 |
| Mixed functions without PHI results | 2 | 3 | 2 |
| Unmatched | 62 | 46 | 49 |
| Sum | 272 | 192 | 194 |

**Table S9.** Functional category statistics of genes located at scaffolds 17, 19, 20 and 22 of *Colletotrichum scovillei* strains Coll-524, Coll-153 and Coll-365.

| Categories | Coll-524/Coll-153/Coll365 | | | | Removed in Coll-153 and /or Coll-365 |
| --- | --- | --- | --- | --- | --- |
|  | S17 | S19 | S20 | S22 | S17+S19+S20+S22 |
| Effector | 20/19/19 | 11/0/0 | 19/0/1 | 2/0/0 | 33 |
| CAZyme | 8/8/7 | 0/0/0 | 0/0/0 | 0/0/0 | 1 |
| SMURF | 0/0/0 | 7/0/0 | 0/0/0 | 0/0/0 | 7 |
| TF | 6/4/4 | 1/0/0 | 3/0/0 | 1/1/0 | 7 |
| KEGG | 10/10/9 | 2/1/0 | 4/0/0 | 0/0/0 | 7 |
| PHI |  |  |  |  |  |
| Increased virulence | 7/7/7 | 4/1/0 | 3/0/0 | 1/0/0 | 8 |
| Reduced virulence | 61/61/54 | 21/0/0 | 42/3/2 | 11/0/0 | 79 |
| Loss of pathogenicity | 8/7/6 | 6/0/0 | 5/1/0 | 0/0/0 | 12 |
| Lethal | 4/4/4 | 2/0/0 | 3/0/0 | 1/0/0 | 6 |
| Mixed results | 8/8/8 | 2/0/0 | 6/0/0 | 1/0/0 | 9 |

**Table S10.** Repeat sequence analysis with MISA for microsatellite compositions in the three strains.

| Results of microsatellite search | Coll-524 | Coll-153 | Coll-365 |
| --- | --- | --- | --- |
| Total number of scaffolds examined | 54 | 59 | 59 |
| Total number of identified SSRs | 6369 | 6290 | 6261 |
| Number of SSR containing sequences | 47 | 48 | 49 |
| Number of sequences containing more than 1 SSR | 43 | 41 | 44 |
| Number of SSRs present in compound formation | 433 | 423 | 426 |

**Table S11.** The distribution of the 59 genes clustered at different scaffolds in strain Coll-524. The numbers of genes absent in both strains Coll-153 and Coll-365, and in the strain Coll-365 only, are presented.

| Scaffold | No. of genes absent in  Coll-153  and Coll-365 | No. of genes absent in  Coll-365 |
| --- | --- | --- |
| 4 | 0 | 1 |
| 5 | 1 | 0 |
| 8 | 1 | 0 |
| 10 | 1 | 0 |
| 12 | 1 | 0 |
| 14 | 0 | 1 |
| 15 | 3 | 0 |
| 16 | 1 | 0 |
| 17 | 0 | 5 |
| 19 | 18 | 1 |
| 20 | 18 | 4 |
| 22 | 3 | 0 |
| Sum | 47 | 12 |

**Table S12.** Pathogenicity assay of transgenic strains and wild-type strain Coll-365 (WT) on fruits of *Capsicum annuum* cv. Groupzest by pair inoculation.

| Strains | N^a^ | Mean of lesion size (mm^2^) | | *P* value |
| --- | --- | --- | --- | --- |
|  |  | WT | Transgenic |  |
| X-1 | 2 | 7.80 | 105.70 | 0.0130 |
| X-2 | 3 | 5.60 | 48.60 | 0.0370 |
| VI-1 | 4 | 30.82 | 24.00 | 0.2740 |
| VI-2 | 3 | 34.43 | 22.71 | 0.1382 |

^a^, N indicates the numbers of fruits used in each inoculation.

**Table S13.** TransposonPSI analysis results of three *Colletotrichum scovillei* strains and *C. higginsianum* IMI 349063.

| **Transposable element** | **Coll-524** | **Coll-153** | **Coll-365** | ***C. higginsianum* IMI 349063** |
| --- | --- | --- | --- | --- |
| gypsy | 136 | 133 | 131 | 172 |
| DDE_1 | 106 | 133 | 35 | 471 |
| mariner_ant1 | 84 | 0 | 0 | 166 |
| cacta | 23 | 23 | 23 | 42 |
| LINE | 17 | 16 | 14 | 49 |
| TY1_Copia | 8 | 8 | 8 | 93 |
| helitronORF | 6 | 5 | 5 | 7 |
| MuDR_A_B | 6 | 6 | 6 | 12 |
| mariner | 4 | 2 | 2 | 8 |
| P_element | 2 | 0 | 0 | 0 |
| ltr_Roo | 1 | 1 | 1 | 3 |
| piggybac | 1 | 0 | 0 | 2 |
| Sum | 394 | 327 | 225 | 1025 |
